# Engineering double-stranded RNA binding activity into the Drosha double-stranded RNA binding domain results in a loss of microRNA processing function

**DOI:** 10.1371/journal.pone.0182445

**Published:** 2017-08-08

**Authors:** Joshua C. Kranick, Durga M. Chadalavada, Debashish Sahu, Scott A. Showalter

**Affiliations:** 1 Department of Chemistry, Center for RNA Molecular Biology, The Pennsylvania State University, University Park, Pennsylvania, United States of America; 2 Department of Biochemistry and Molecular Biology, The Pennsylvania State University, University Park, Pennsylvania, United States of America; University of Hong Kong, HONG KONG

## Abstract

Canonical processing of miRNA begins in the nucleus with the Microprocessor complex, which is minimally composed of the RNase III enzyme Drosha and two copies of its cofactor protein DGCR8. In structural analogy to most RNase III enzymes, Drosha possesses a modular domain with the double-stranded RNA binding domain (dsRBD) fold. Unlike the dsRBDs found in most members of the RNase III family, the Drosha-dsRBD does not display double-stranded RNA binding activity; perhaps related to this, the Drosha-dsRBD amino acid sequence does not conform well to the canonical patterns expected for a dsRBD. In this article, we investigate the impact on miRNA processing of engineering double-stranded RNA binding activity into Drosha’s non-canonical dsRBD. Our findings corroborate previous studies that have demonstrated the Drosha-dsRBD is necessary for miRNA processing and suggest that the amino acid composition in the second α-helix of the domain is critical to support its evolved function.

## Introduction

MicroRNAs (miRNAs) are a class of small (20–22 nt) non-coding RNAs known to function primarily in the cellular process of RNA silencing [1]. In order to execute its role in post-transcriptional gene regulation, a mature miRNA binds a complementary messenger RNA (mRNA) in the RNA induced silencing complex (RISC) and inhibits translation of the target mRNA [2, 3]. Through their silencing functions, mature miRNAs have been shown to exert control over many essential biological functions, including regulation of the cell cycle and cellular differentiation [4, 5]. Among other broad applications, this has resulted in intense study of the role miRNAs play in tumor cell proliferation, innate immunity, and other disease mechanisms [6–8]. It is therefore no surprise that there is also a parallel and vigorous effort to understand the molecular mechanism of miRNA maturation.

The canonical miRNA maturation pathway begins with a primary transcript (pri-miRNA, often encoded within the introns of nascent mRNA transcripts) that is processed in the nucleus via the Microprocessor complex, which is comprised minimally of the catalytic unit, Drosha, and two copies of its cofactor, DGCR8 [9]. After this initial processing, the precursor miRNA (pre-miRNA) is exported from the nucleus via the Exportin-5 pathway [10] and further processed in the cytosol by a complex minimally composed of Dicer, and its cofactor TRBP (or in some cases PACT). This complex then associates with an Argonaut protein for transfer of the miRNA guide strand into RISC [2, 11], where regulation of its complimentary mRNA(s) occurs.

**Fig 1:**
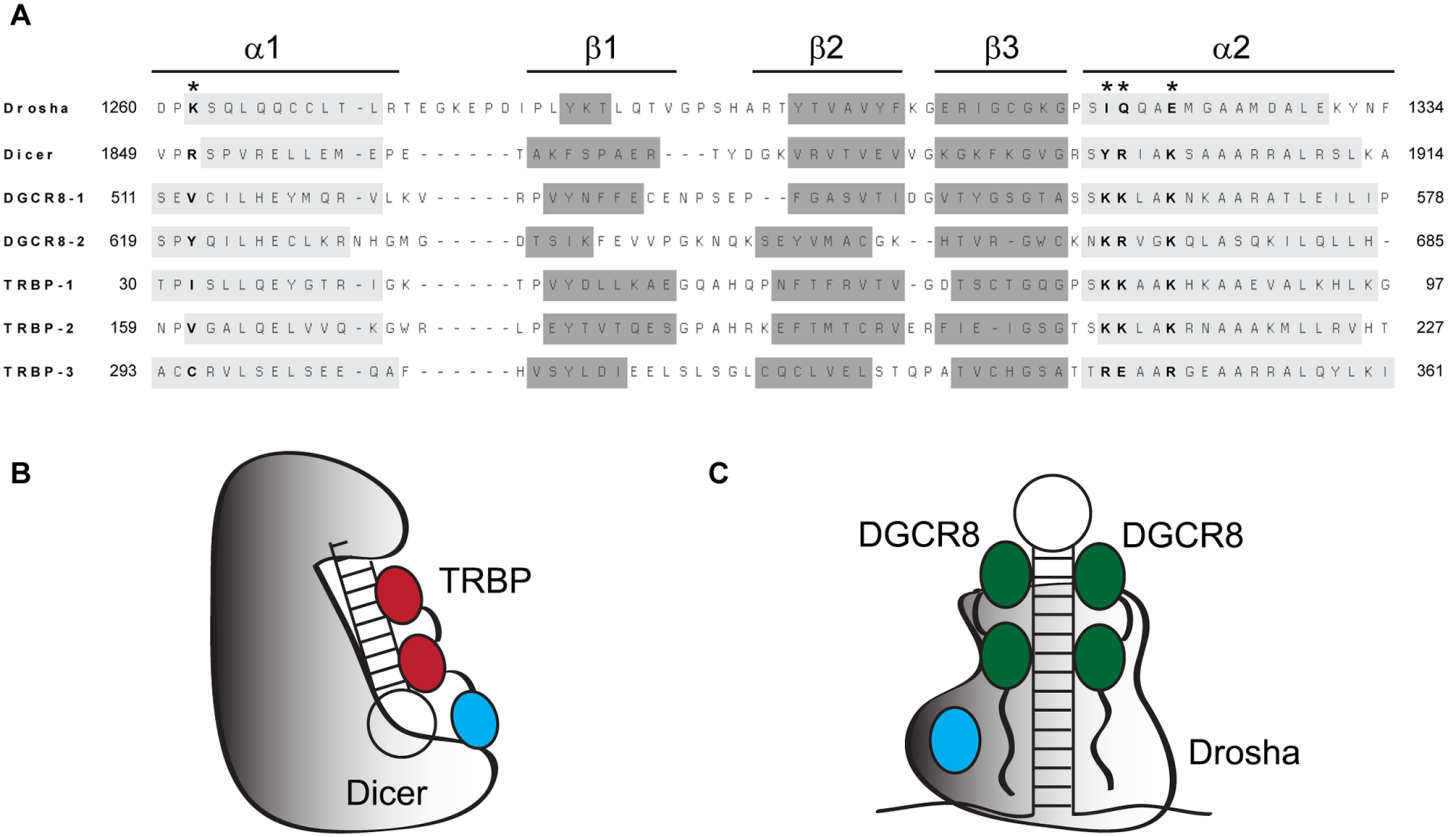
Conservation of sequence and secondary structure in the dsRBDs of miRNA maturation pathway components. (A) Sequence alignment of miRNA maturation pathway dsRBDs, with residue numbers provided relative to the human polypeptide sequence. Secondary structures present in the known crystallographic or NMR structure of each domain are annotated in shaded boxes within individual sequences, with general boundaries annotated below. Secondary structure assignments correspond to the following PDB files: Drosha, 2KHX; [19] Dicer, 3C4B; [20] DGCR8-1 and DGCR8-2, 2YT4;[21] TRBP-1, 3LLH (unpublished); TRBP-2, 3ADL;[22] and TRBP-3, 4WYQ.[17] Asterisks and bolded text are used to annotate the residues mutated to generate the Drosha-Quad mutant. (B) Schematic depicting the pre-RISC Dicer/TRBP/pre-miRNA complex, modelled to reflect interactions between TRBP’s dsRBDs 1 and 2 (Red) and the stem of the pre-miRNA. TRBP-dsRBD3 (Blue) mediates protein-protein interactions with Dicer (grey scale), but has no known dsRNA binding function. (C) Schematic depiction of the Microprocessor complex modelling the observation that DGCR8 (dsRBDs green ovals, disordered regions black wavy lines) recognizes the distal loop while the catalytic unit, Drosha (grey scale, dsRBD blue), recognizes and processes the apical stem. Similar to TRBP-dsRBD3, Drosha-dsRBD has no known dsRNA binding function.

Double-stranded RNA binding domains (dsRBDs) are present in each major protein in the miRNA maturation pathway (with the exception of Exportin-5), making them likely candidate domains for RNA substrate recognition. Each dsRBD fold is comprised of a strictly conserved α-β-β-β-α structure; sequence is not necessarily conserved, although some consensus elements have been identified [12]. Both RNase III enzymes found in the miRNA processing pathway, Drosha and Dicer, contain a single C-terminal dsRBD, whereas DGCR8 and TRBP each contain multiple dsRBDs. The canonical binding face for dsRBDs includes protein residues found in three spatially distinct regions initially identified by Ryter and Shultz [13]. Of greatest relevance to the present study is Region 3: a cluster of residues at the N-terminus of α2 that is enriched in arginine and lysine residues. Significantly, the presence of the Region 3 sequence motif is not strictly conserved throughout the pathway. For example, the Region 3 motif is not present in dsRBD3 of TRBP and PACT, or in Drosha’s dsRBD (Fig 1). Significantly, experimental evidence demonstrates that the third dsRBDs of TRBP and PACT function as mediators of protein-protein interactions, not as mediators of dsRNA binding [14–16]. Interest in the non-RNA binding dsRBDs of the miRNA processing pathway was recently elevated by a co-crystal structure of TRBP-dsRBD3 in complex with the protein binding domain (PBD) of Dicer [17]. Although Drosha’s dsRBD is essential for miRNA processing [9], we have shown that it does not bind to dsRNA *in vitro* [18]. The uniform absence of dsRNA binding activity among these three dsRBDs that possess non-canonical Region 3 motif sequences suggests a causative relationship that has not yet been explored.

Here we present our success in engineering a dsRNA binding Drosha-dsRBD mutant, which was achieved through site directed mutagenesis of the Region 3 motif. We refer to the generated construct throughout as “Drosha-Quad,” in acknowledgement of the need for four point mutations to encode the reported gain of function. Biophysical confirmation that the overall fold was retained by solution NMR spectroscopy and demonstration that its RNA binding activity is similar to that of other canonical dsRBDs in the miRNA processing pathway. Significantly, cell-based pri-miRNA processing assays conducted in the background of the Drosha-Quad mutations demonstrate that avid dsRNA binding by the dsRBD of Drosha is incompatible with efficient pri-miRNA processing.

## Materials and methods

### Protein preparation

Mutants were generated by the standard QuikChange Lightning site-directed mutagenesis protocol,(Agilent Technologies) using a previously reported plasmid that encodes wt-Drosha-dsRBD (1259–1337) in a pET-47b(+) vector [18]. Upon sequence confirmation, the mutant plasmid was transformed into the *Escherichia coli* BL21(DE3) cell line for overexpression. Cells were grown to OD_600_ = 0.5 at which point they were induced with 500 μM IPTG (final concentration) at 24°C for 16 hours. Cells were lysed via sonication and the expressed protein was purified as previously described for wt-Drosha-dsRBD [18], although the final experimental buffers were modified as described below.

### RNA preparation

In preparation for both the binding and processing assays, pri-mir-16-1 was transcribed *in vitro*, purified, and renatured as previously described [18]. For the long ds44 and ds33 constructs, *in vitro* transcription using a duplex method, followed by purification, was performed as previously described [23]. The shorter ds22 RNA strands were purchased from Dharmacon and deprotected using the manufacturer protocol prior to duplex formation. The RNA sequences for both strands of each duplex used in this study are presented in Table 1.

**Table 1:** List of RNA sequences used in this work.

Name	RNA sequence 5´ to 3´
pri-mir-16-1	GGG UGA UAG CAA UGU CAG CAG UGC CUU AGC AGC ACG UAA AUA UUG GCG UUA AGA UUC UAA AAU UAU CUC CAG UAU UAA CUG UGC UGC UGA AGU AAG GUU GAC CAU ACU CUA C
ds44 TS	GGU CAG CAG UGC CUU AGC AGC ACG UAA AUA UUG GCG UUA AGA CC
ds44 BS	GG UCU UAA CGC CAA UAU UUA CGU GCU GCU AAG GCA CUG CUG ACC
ds33 TS	GGU CAG CAG UGC CUU AGC AGC ACG UAA AUA UGG
ds33 BS	CCA UAU UUA CGU GCU GCU AAG GCA CUG CUG ACC
ds22 TS	GUC AGC AGU GCC UUA GCA GCA C
ds22 BS	G UGC UGC UAA GGC ACU GCU GAC

### NMR methods

Samples of uniformly isotope enriched ^15^N-Drosha-Quad or ^15^N,^13^C-Drosha-Quad were prepared as described to a final concentration of ~800 μM protein in 100 mM sodium cacodylate, pH 7.3, 100 mM KCl, 5 mM β-mercaptoethanol, with 10% (v/v) D_2_O to provide a lock signal. All NMR spectra were collected at 25°C.

Drosha-Quad backbone and side-chain resonances were assigned using standard ^1^H,^15^N-HSQC, ^1^H,^13^C-HSQC, HNCO, HN(CA)CO, CBCA(CO)NH, HNCACB, H(CCCO)NH, and (H)CC(CO)NH experiments as implemented in the Topspin 3 pulse program library. An aliphatic TOCSY mixing time of 22 ms was used for side-chain assignments. Additional side-chain assignments were made using the standard HCCH-TOCSY experiment with a 36 ms mixing time. All experiments were performed on a Bruker Avance III 600 MHz spectrometer equipped with a TCI cryoprobe for enhanced sensitivity. All triple resonance experiments were performed using 25–40% sparsity non-uniform sampling. Spectra were processed in Topspin 3 (Bruker) and analyzed using Sparky (T. D. Goddard and D. G. Kneller, SPARKY 3, University of California, San Francisco). Chemical shift data for Drosha-Quad has been deposited in the BioMagResBank (ascension number 25924).

### ModelFree analysis

The dynamic properties of Drosha-Quad were examined using Lipari-Szabo model-free analysis [24] as implemented in the ModelFree 4.20 software [25]. The diffusion tensor used in these calculations was calculated using the quadric method [26, 27]. Internal dynamics for all residues were described by fitting 600 MHz R_1_, R_2_ and NOE relaxation data to determine *S*^*2*^ and *τ*_*e*_ as previously described [18].

### EMSA methods

In preparation for binding assays involving the variable length duplexes, the top strand RNA was 5´-end labelled with γ-^32^P-ATP and mixed in a 3-fold molar excess with cold bottom strand. The duplex was purified from an 8% acrylamide native gel. The pri-mir-16-1 construct was similarly labeled with ^32^P-ATP and renatured via heating at 90°C for 1 minute and snap cooling at 4°C for 5 min. Binding reactions were run in 50 mM sodium cacodylate pH 6.0, 50 mM KCl, 5% glycerol, 1mM dithiothreitol, 0.1 mg/ mL Bovine Serum Albumin and 0.1 mg/ mL herring sperm DNA for thirty minutes at room temperature. The reactions were loaded onto a 10% acrylamide native gel and run at 200 V for 3.5 hrs at 4°C.

Gels were imaged using a Typhoon-9410 imager and then quantified using the software ImageQuant (GE Healthcare Life Sciences) as previously described [28]. For each lane, ImageQuant was used to draw equivalent sized boxes around the unbound band and the space between the unbound band and the bottom of the well. The fraction of RNA bound was determined by computing the ratio of the integrated intensities inside the two boxes for each lane. The points in the titration curves represent the mean fraction bound from two gels and the error indicated is the uncertainty of this value to one standard deviation. The curves were fit to the general Hill equation binding model using Matlab (MathWorks).

### Processing assay methods

All Drosha constructs and WT DGCR8 were overexpressed in HEK-293T cells. Plasmid was transfected into cells using Lipofectamine 2000 (Invitrogen) per the packaged instructions. Mock was similarly treated with Lipofectamine using the same procedure, but lacking plasmid DNA. Forty-eight hours later the cells were washed and subsequently harvested using phosphate-buffered saline and lysed via sonication using a buffer comprised of 20mM Tris, 100 mM KCl and 0.2 mM EDTA at pH 8.0. The lysate was cleared of cellular debris via centrifugation and combined with RNasin (Promega), 10 fmol 5´-end labeled RNA was generated using γ-^32^P-ATP as described above, or body labeled using α-^32^P-UTP as previously described [28]. For all reactions, MgCl_2_ was added to a final concentration of 6.4 mM. The reactions were incubated at 37°C for 30 minutes and immediately thereafter loaded onto 10% polyacrylamide denaturing gels for analysis. Processing efficiency was calculated by comparing the density of the pri-miRNA band to that of the 5´-fragment (end labeled) or pre-miRNA band (body labeled), with uncertainties in the relative densities determined from the standard deviation of triplicate measurements.

## Results

### Generation of the Drosha-Quad mutant

**Fig 2:**
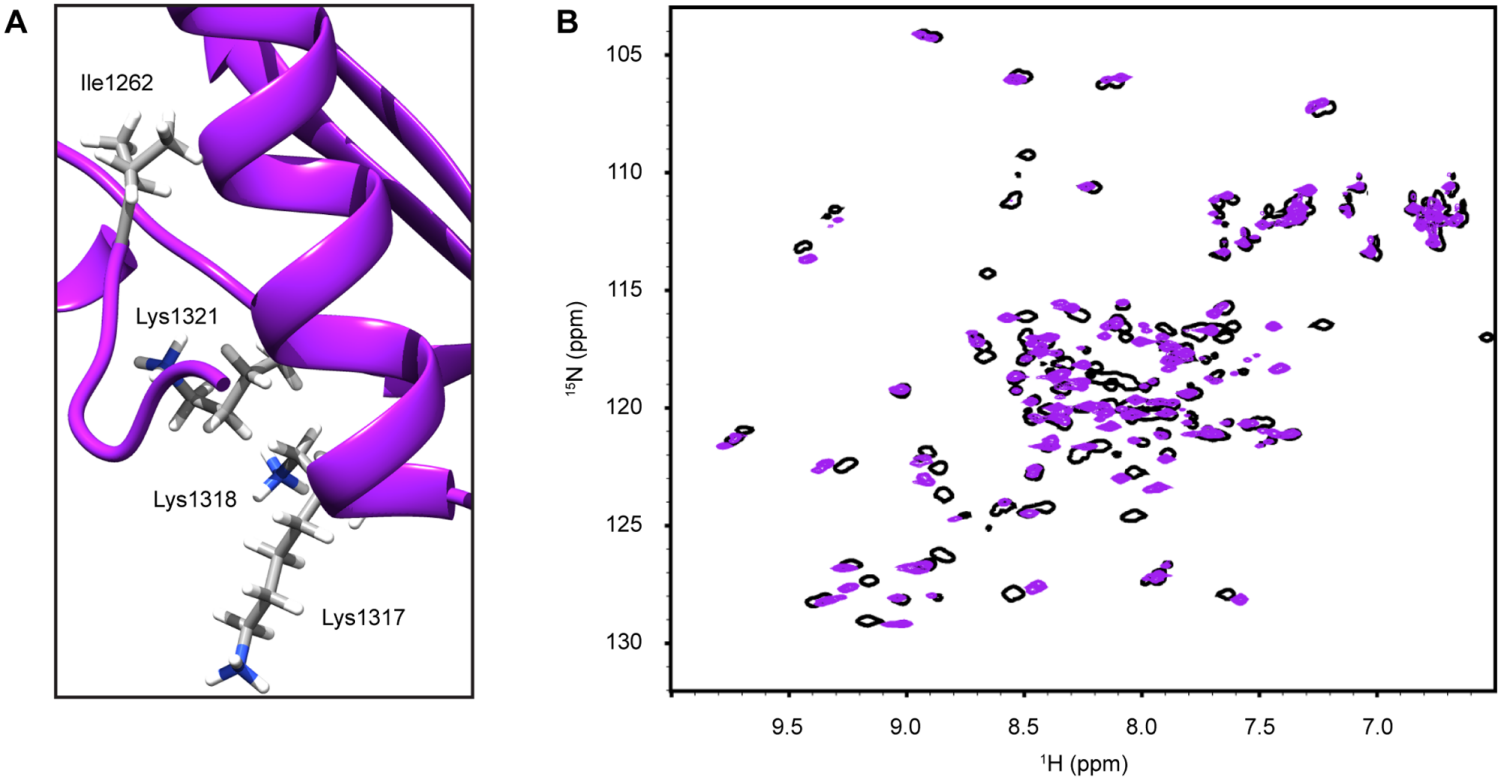
NMR analysis of Drosha-Quad structure reveals preservation of the dsRBD fold with a well-packed Region 3 motif. (A) Tertiary packing near Region 3 provides a rationale for protein instability prior to introduction of the K1262I mutation in α1. The four mutated residues are shown in stick representation on the ribbon diagram. (B) ^1^H,^15^N-HSQC overlay of wt-Drosha-dsRBD (hollow black) and Drosha-Quad (purple) confirms good peak dispersion, characteristic of a folded structure, and an overall similarity to the wild-type HSQC.

The three dsRBDs found in the miRNA processing pathway that do not possess demonstrated dsRNA binding activity all have non-canonical amino acid sequences in their Region 3 motif (Fig 1). Therefore, this study was initiated through mutagenesis of Drosha-dsRBD designed to directly test the hypothesis that the dsRNA binding function of dsRBDs is established by the amino acid composition of the Region 3 motif. Re-introduction of the Region 3 motif into Drosha-dsRBD through three point mutations (I1317K, Q1318K, E1321K; numbering relative to the human polypeptide sequence) resulted in an insoluble recombinant construct when expressed in *E*. *coli*. Examination of the protein sequence, compared to other dsRBDs (Fig 1) revealed that an unusual lysine residue is present near the N-terminus of helix-1 (marked with an asterisk in the sequence alignment). Visual inspection of the NMR structure of wild-type Drosha-dsRBD demonstrated that this lysine projects into the same region of space as the three sidechains from the Region 3 motif,[19] leading us to speculate that too much charge was being introduced into this region in our mutant, causing the domain to become unstable. Mutation of this residue to a β-branched aliphatic amino acid (K1262I), which is more representative of the canonical dsRBD sequence motif, in the background of the three α2 mutations already introduced, yielded the soluble and stable recombinant construct (Drosha-Quad) reported here (visualized in Fig 2A).

**Fig 3:**
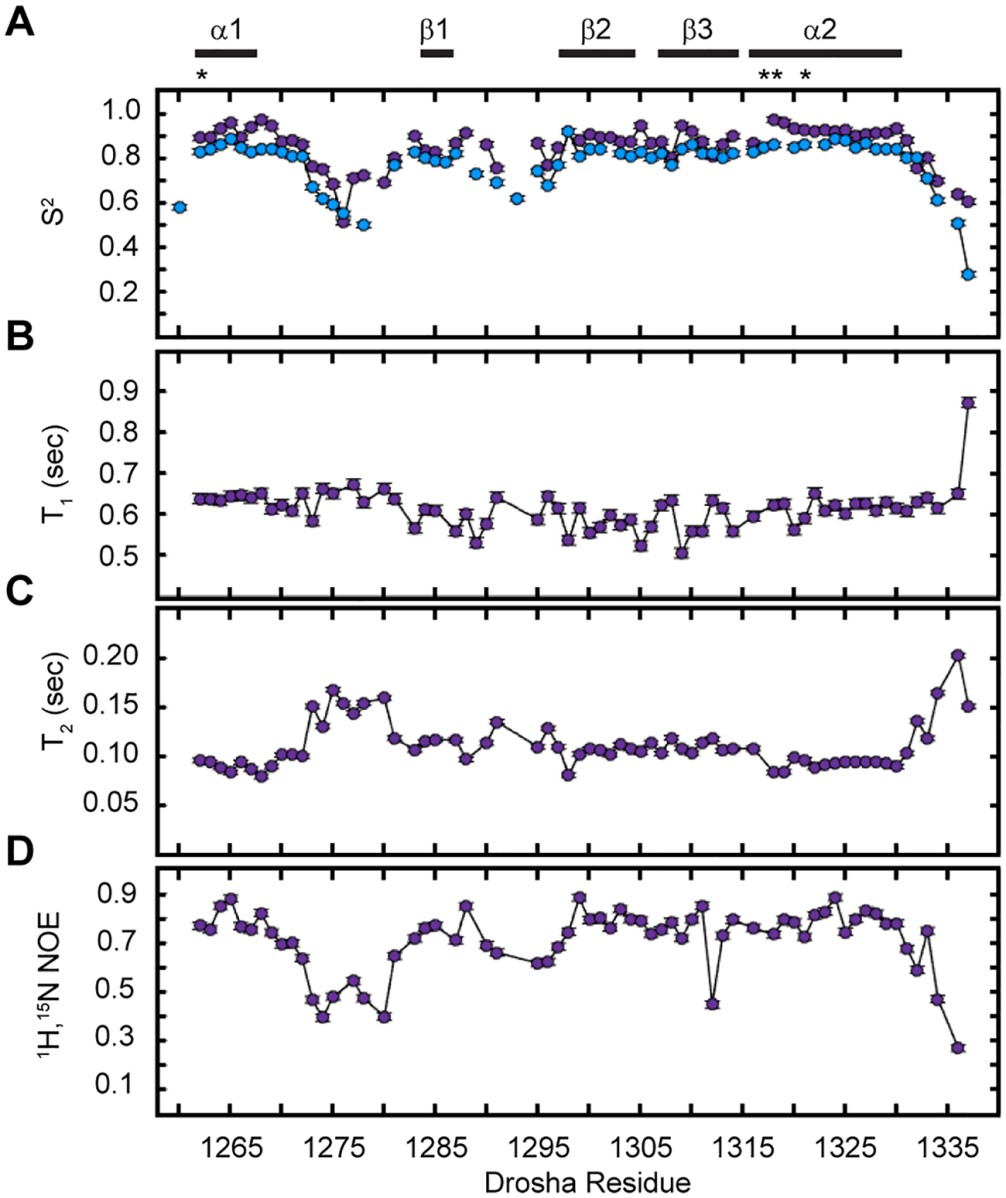
NMR spin relaxation reveals similar backbone structural dynamics in wt-Drosha-dsRBD and Drosha-Quad. (A) Per-residue order parameters (S^2^) for wt-Drosha-dsRBD (blue) and Drosha-Quad (purple). Uncertainties in the best fit value of S^2^ are represented as error bars that are generally smaller than the size of the marker. Order parameters were derived by fitting per-residue (B) longitudinal relaxation times (T_1_ = 1/R_1_), (C) Transverse relaxation times (T_2_ = 1/R_2_), and (D) [^1^H]-^15^N NOE data collected at 600 MHz proton resonant frequency. Secondary structure positions are annotated with bars above the figure and the positions of the four mutations introduced to generate Drosha-Quad are indicated with an asterisk.

Analysis of the HSQC overlay, comparing WT Drosha-dsRBD and Drosha-Quad (Fig 2B), reveals excellent signal dispersion and overall preservation of the wild-type resonance pattern [18]. We next aimed to confirm that the backbone dynamics of these two constructs are also consistent through measurement of ^15^N-spin relaxation (Fig 3). Reassuringly, qualitative trends in the calculated order parameters for wt-Drosha-dsRBD and Drosha-Quad are nearly identical (Fig 3A) and both are consistent with estimates of ps-ns dynamics established by [^1^H],^15^N-NOE values independently reported for Drosha-dsRBD [19]. While there is a slight baseline offset in the order parameter profiles, this may be attributable to subtle changes in the diffusion tensor for Drosha-Quad and therefore is not deemed large enough to be significant. It is noteworthy that the order parameters in the mutated portion of α2 (i.e., Region 3) are among the most different, suggesting that some stiffening of the backbone may have occurred as a result of the mutations. Taken together, these NMR data confirm that the overall structure and dynamics of Drosha-Quad are in good agreement with those of the wild type domain.

### Region 3 sequence drives dsRNA binding by Drosha-dsRBD

In general, dsRBD proteins bind to A-form dsRNA duplexes with observed equilibrium dissociation constants in the low micromolar range (K_d,obs_ ~ 1–10 μM) under *in vitro* conditions and do so without (strong) sequence preference,[29, 30] although exceptions that display sequence specificity have been noted [31, 32]. Our laboratory has characterized the structure of an *in vitro* transcribed pri-mir-16-1 construct [33] and used this model to establish the dsRNA binding affinity of the dsRBDs from DGCR8 [18, 28], TRBP [34], and Dicer [23], as summarized in Table 2. Our results have proven to be highly consistent with those reported by other laboratories using related pri-miRNA constructs [35, 36].

**Table 2:** Best fit observed macroscopic binding affinities (K_d,obs_, μM) as determined by EMSA for dsRBD constructs relevant to the miRNA processing pathway.

RNA	Drosha-Quad	Dicer-dsRBD^a^	DGCR8-dsRBD1^b^	TRBP-dsRBD1^c^	TRBP-dsRBD2^c^
pri-mir-16-1^d^	2.8 ± 0.1	2.2 ± 0.1	9.7 ± 0.6	N/D^e^	N/D^e^
ds44	6.5 ± 0.6	2.4 ± 0.1	5.9 ± 0.1	0.8 ± 0.05	0.8 ± 0.06
ds33	7.8 ± 0.2	4.9 ± 0.1	8.8 ± 0.2	0.9 ± 0.3	1.0 ± 0.08
ds22	13.4 ± 0.4	6.5 ± 0.1	21 ± 1.0	3.5 ± 0.2	1.7 ± 0.1

^a^ Data reproduced from [23].

^b^ Data reproduced from [18].

^c^ Data reproduced from [34].

^d^ pre-mir-16-1 was substituted for interaction with Dicer-dsRBD.

^e^ Binding not determined.

**Fig 4:**
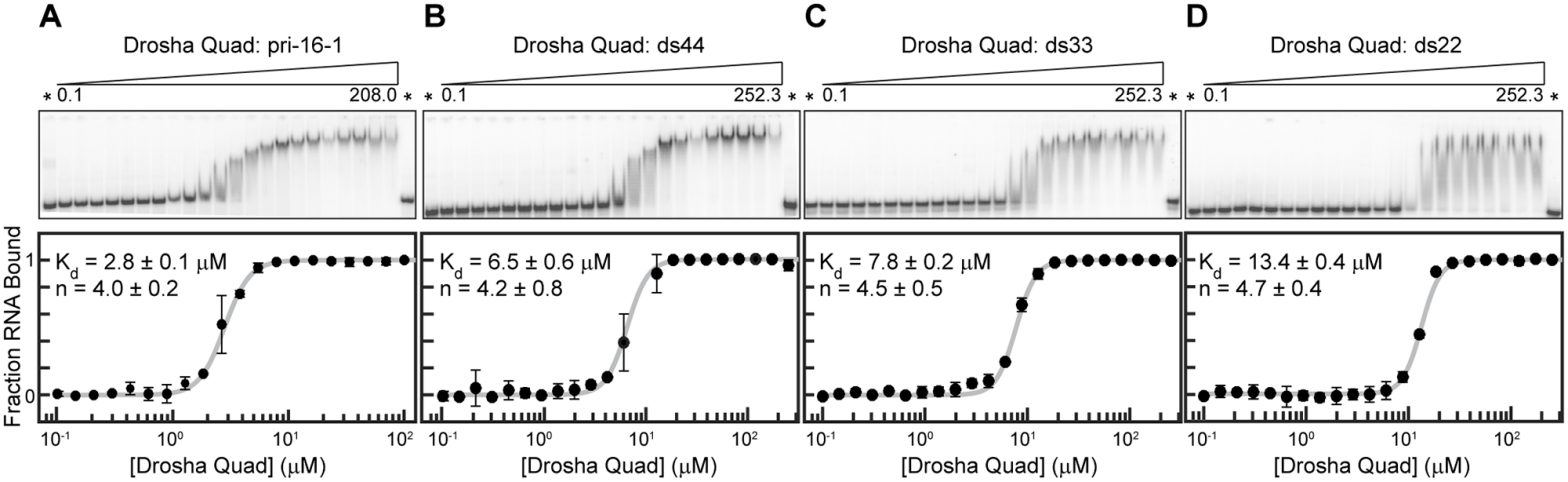
Electrophoretic mobility shift assays of Drosha-Quad binding to a panel of dsRNA substrates. (A) Drosha-Quad titrated into an *in vitro* model for pri-mir-16-1, (B) ds44, (C) ds33, and (D) ds22 perfect Watson-Crick duplexes. Asterisks denote RNA only lanes and increasing protein concentration ranges are indicated below the wedge. In the bottom panels the fraction bound in each lane is displayed as average ± standard deviation, with fits to the general Hill Equation presented as a solid grey line. The insets provide averaged best fit values of the observed dissociation constant (K_d_) and Hill coefficient (n) with errors to one standard deviation.

Although wt-Drosha-dsRBD possesses no native dsRNA binding activity,[18] we observe strong binding to pri-mir-16-1 by Drosha-Quad (K_d,obs_ = 2.8 ± 0.1 μM; Table 2) through native gel assays (Fig 4A). Beyond characterizing the equilibrium binding of native-inspired pri-miRNA constructs, our laboratory has also established that isolated dsRBDs characteristically bind to longer dsRNA duplexes with modestly tighter observed affinities when length is varied in duplexes of <50 base-pair total length (Table 2). For comparison, the stem region of pri-mir-16-1 contains 39 base pairs. Although the measured equilibrium constants for Drosha-Quad binding to the same panel of dsRNA duplexes are on the high end of our previously reported range (Table 2; see Fig 4B–4D for representative EMSA gels and fits), the same trend of tighter binding to longer dsRNA constructs is observed. Taken together, we conclude from this data that modifying the amino acid sequence of the Drosha-dsRBD Region 3 motif to be near-canonical is sufficient to restore native-dsRBD binding behavior to the domain.

### Preserving the wt-Drosha-dsRBD Region 3 motif is essential for miRNA processing

**Fig 5:**
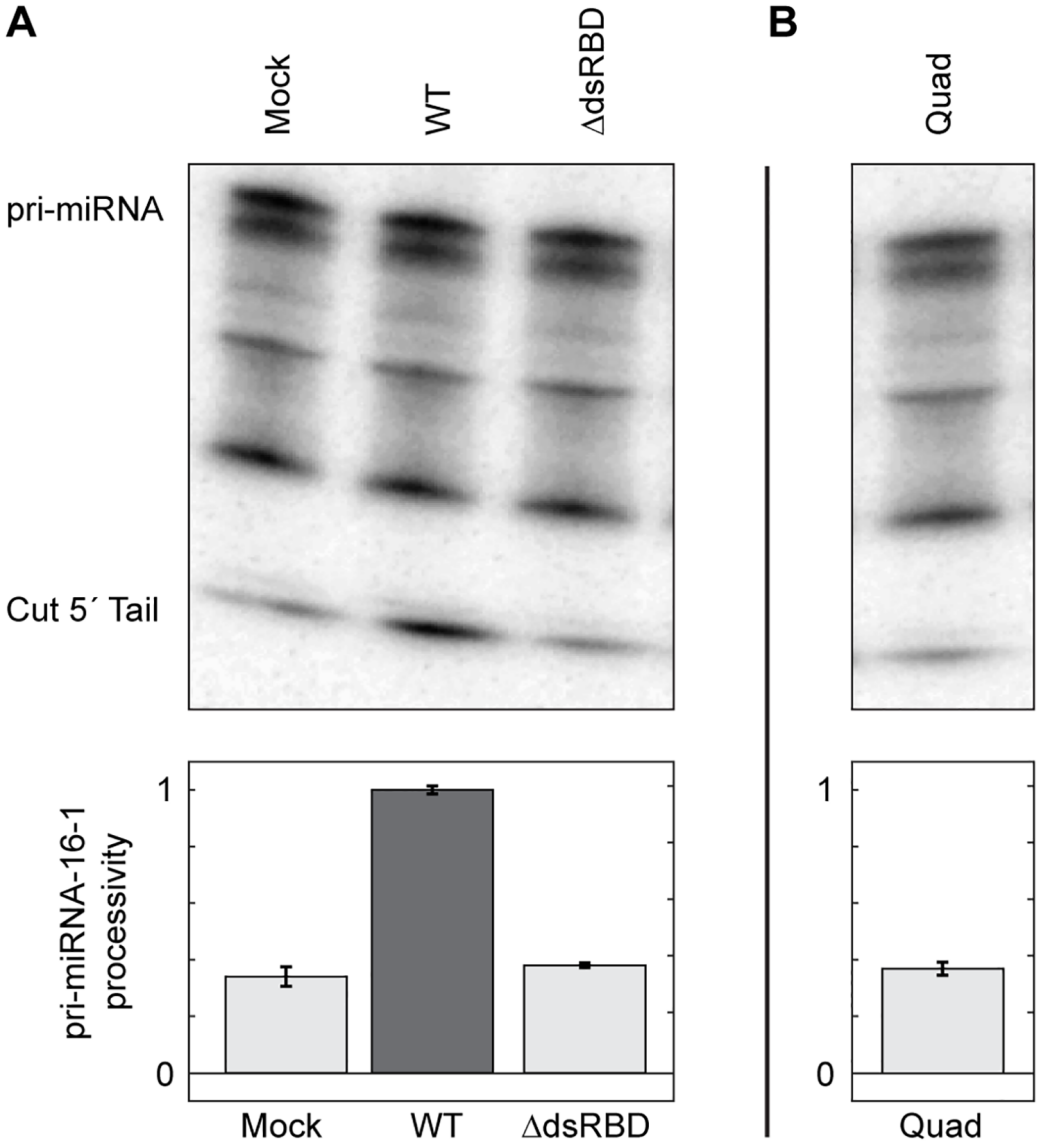
HEK 293T cell-based processing assays reveal that Drosha-Quad is unable to stimulate miRNA processing. (A) A representative gel depicting processing of 5´ end-labelled pri-mir-16-1 by recombinant Microprocessor in HEK 293T cell lysate incorporating wt-Drosha or Drosha-ΔdsRBD. (B) The same processing assay performed with Drosha-Quad. In both panels, the migration position of uncut pri-mir-16-1 and the cut 5´ fragment are labelled.

Having established that Drosha-Quad displays canonical dsRNA binding activity, we finished this study by investigating whether or not our altered Drosha-dsRBD supports native miRNA processing. To this end, we incorporated the Drosha-Quad point mutations into full-length Drosha and assayed the resulting protein for processing ability in the context of the Microprocessor. First, in order to verify that the Drosha-dsRBD is necessary for miRNA processing in our assay, we co-transfected 293T cells with plasmids encoding wild-type DGCR8 and Drosha-ΔdsRBD (a construct in which the C-terminal dsRBD was deleted). As shown in Fig 5A, transfection with wt-Drosha yields enhanced processing of our pri-mir-16-1 substrate, whereas transfection with Drosha-ΔdsRBD did not elevate processing above mock levels. Unexpectedly, transfection with a plasmid encoding full-length Drosha-Quad also did not enhance pri-mir-16-1 processing above mock levels (Fig 5B). The results of our assay lead to the conclusion that Drosha-mediated miRNA cleavage activity is incompatible with avid dsRNA binding activity attributable to the Drosha-dsRBD.

**Fig 6:**
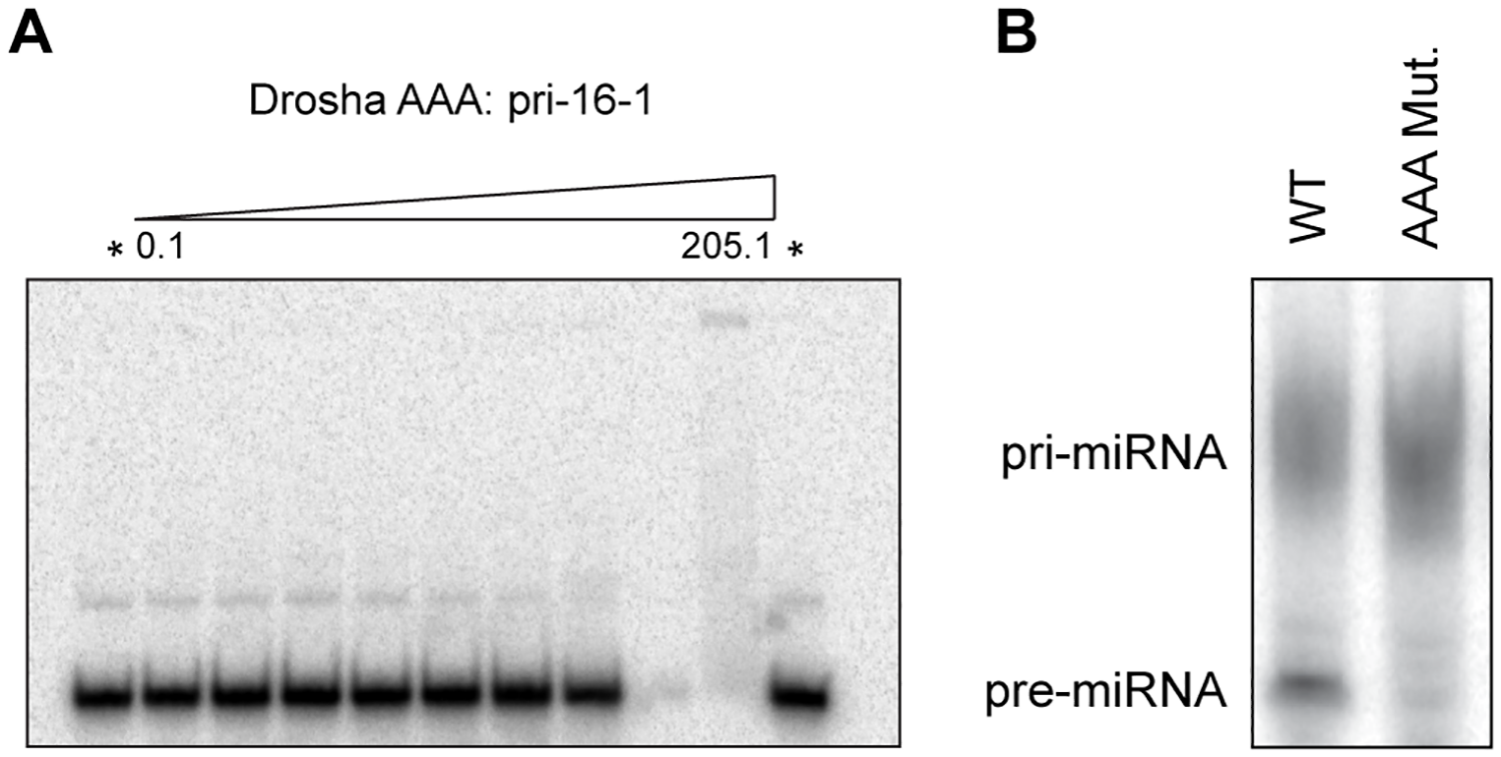
Replacement of critical Region 3 residues with alanine prevents miRNA processing while preserving a lack of dsRNA binding. (A) A representative EMSA gel demonstrating that the triple-alanine Drosha-dsRBD mutant is not able to bind dsRNA at achievable protein concentrations. (B) A representative gel depicting processing of uniformly α-^32^P-ATP body-labelled pri-mir-16-1 by recombinant Microprocessor in HEK 293T cell lysate. The migration positions of uncut pri-mir-16-1 and processed pre-mir-16-1 are labelled.

While compelling, the Drosha-Quad processing results do not address whether dsRNA binding activity is directly responsible for the observed loss of processing function, or if the non-canonical amino acid composition of Region 3 has arisen in support of another unidentified function, such as mediation of protein-protein interactions. We and others have previously shown with DGCR8 [21] and TRBP [34] that alanine-substitution mutation of the Region 3 motif is sufficient to eliminate dsRNA binding activity in dsRBDs. Therefore, as a control to test the hypothesis that impaired miRNA processing by Drosha-Quad is a result of altered Region 3 sequence, and not directly a result of gain-of-function with respect to dsRNA binding, we introduced three alanine substitution mutations (I1317A, Q1318A, and E1321A). This triple-alanine Drosha-dsRBD mutant was confirmed through EMSA not to bind dsRNA (Fig 6A). Significantly, transfection of 239T cells with full-length Drosha bearing the triple-alanine elimination of wild-type Region 3 sequence failed to support pri-mir-16-1 processing (Fig 6B). In summary, disruption of the Region 3 sequence in Drosha-dsRBD is sufficient to inhibit miRNA processing in cellular extracts, regardless of whether the mutant dsRBD displays dsRNA binding activity or not.

## Discussion

Recent characterization of the Dicer-PBD:TRBP-dsRBD3 interaction [17] has stimulated new interest in understanding the function of non-canonical dsRBDs in the context of the miRNA processing pathway. While the non-canonical dsRBD of Drosha and TRBP-dsRBD3 are similar in their demonstrated lack of dsRNA binding activity, the data presented here provides an intriguing contrast with the mechanism of TRBP-dsRBD3 function. In the case of TRBP, the Region 3 motif region of the dsRBD surface is not part of the protein-protein interface formed with Dicer. In contrast, we have shown that the Drosha-dsRBD function in the context of the Microprocessor depends on preservation of its wild-type, non-canonical Region 3 motif sequence. This observation suggests that the necessary function of Drosha-dsRBD in miRNA processing is likely to be mediated by macromolecular interactions that are structurally dissimilar to the TRBP-Dicer interaction.

As the example of TRBP-dsRBD3 illustrates, the dsRBD fold is not exclusively used in nature for dsRNA binding, but rather many variations have evolved to support other functions, often mediated by protein-protein interactions. For example, insertion of extra residues into the α1-β1 or the β1-β2 loop of the dsRBD fold has previously been suggested as a mechanism for imparting dual RNA and protein binding activity [37, 38]. As seen in Fig 1, Drosha-dsRBD contains an extension of this type. This suggests that protein-protein interactions may be mediated by the extended α1-β1 loop of Drosha-dsRBD too. We have previously suggested that the dynamics of Drosha-dsRBD’s extended α1-β1 loop may in some way impair dsRNA binding by the domain [18]. In contrast, our present work shows convincingly that the loss of non-canonical but conserved Region 3 motif sequence in this dsRBD is responsible for its loss of dsRNA binding activity.

Finally, the advances we report here in understanding Drosha-dsRBD function are especially intriguing in light of recent work that has challenged a number of previous DGCR8-centric models for Microprocessor assembly. In a landmark study, the Kim lab has shown that the minimal Microprocessor components needed to reconstitute function *in vitro* are the C-terminal region of Drosha, from the central domain to the native C-terminus, and a 73-residue C-terminal tail of DGCR8 [39]. It is noteworthy that no specific function was attributed to the C-terminal tail of DGCR8 in that study [39], although mediation of protein-protein interactions with Drosha is an obvious hypothesis. In both pulldown assays and NMR titrations we were unable to demonstrate an interaction between the C-terminal tail of DGCR8 and the dsRBD from Drosha (data not shown). Therefore, if the functional role of the Drosha-dsRBD in vivo is to mediate protein-protein interactions in the Microprocessor complex, the target(s) of this interaction remain unidentified.

In summary, the C-terminal dsRBD of Drosha deviates from the expected sequence for a dsRBD in two key ways: it possesses a non-canonical Region 3 motif and an extended α1-β1, both of which have previously been associated with biological functions that are unrelated to dsRNA binding. Here we have designed a canonical Region 3 sequence into the Drosha-dsRBD, with the result that we are able to show *in vitro* dsRNA binding properties for Drosha-Quad that are highly consistent with those of other well-characterized dsRBDs. In contrast, we find that Drosha-Quad does not support miRNA processing in the context of the Microprocessor. This result suggests that Drosha-dsRBD may behave consistently with the growing number of non-canonical dsRBDS that mediate protein-protein interactions using the α1-α2 interface [40]. Finally, our processing assays clearly demonstrate that the Drosha-dsRBD is essential for dsRNA processing *in vitro*, which is consistent with the minimal Microprocessor results discussed above [39]. Taken together, these findings demonstrate an affirmative role for Drosha-dsRBD in Microprocessing and suggest that the chemistry of the canonical dsRNA binding face, dominated by the sequence of the Region 3 motif, is strongly coupled to dsRBD function.

## References

[1] Pratt AJ, MacRae IJ. The RNA-induced silencing complex: a versatile gene-silencing machine. J Biol Chem. 2009;284(27):17897–901. doi: 10.1074/jbc.R900012200 19342379 10.1074/jbc.R900012200 PMC2709356

[2] Liu J, Carmell MA, Rivas FV, Marsden CG, Thomson JM, Song JJ, et al Argonaute2 is the catalytic engine of mammalian RNAi. Science. 2004;305(5689):1437–41. doi: 10.1126/science.1102513 15284456 10.1126/science.1102513

[3] Fabian MR, Sonenberg N, Filipowicz W. Regulation of mRNA translation and stability by microRNAs. Annu Rev Biochem. 2010;79:351–79. doi: 10.1146/annurev-biochem-060308-103103 20533884 10.1146/annurev-biochem-060308-103103

[4] Zhao Y, Srivastava D. A developmental view of microRNA function. Trends Biochem Sci. 2007;32(4):189–97. doi: 10.1016/j.tibs.2007.02.006 17350266 10.1016/j.tibs.2007.02.006

[5] Bartel DP. MicroRNAs: target recognition and regulatory functions. Cell. 2009;136(2):215–33. doi: 10.1016/j.cell.2009.01.002 19167326 10.1016/j.cell.2009.01.002 PMC3794896

[6] Sassen S, Miska EA, Caldas C. MicroRNA: implications for cancer. Virchows Arch. 2008;452(1):1–10. doi: 10.1007/s00428-007-0532-2 18040713 10.1007/s00428-007-0532-2 PMC2151131

[7] Ma F, Xu S, Liu X, Zhang Q, Xu X, Liu M, et al The microRNA miR-29 controls innate and adaptive immune responses to intracellular bacterial infection by targeting interferon-gamma. Nat Immunol. 2011;12(9):861–9.doi: 10.1038/ni.2073 21785411 10.1038/ni.2073

[8] Zhang C. MicroRNomics: a newly emerging approach for disease biology. Physiol Genomics. 2008;33(2):139–47. doi: 10.1152/physiolgenomics.00034.2008 18303086 10.1152/physiolgenomics.00034.2008

[9] Han JJ, Lee Y, Yeom KH, Kim YK, Jin H, Kim VN. The Drosha-DGCR8 complex in primary microRNA processing. Genes Dev. 2004;18(24):3016–27. doi: 10.1101/gad.1262504 15574589 10.1101/gad.1262504 PMC535913

[10] Zeng Y, Cullen BR. Structural requirements for pre-microRNA binding and nuclear export by Exportin 5. Nucleic Acids Res. 2004;32(16):4776–85. doi: 10.1093/nar/gkh824 15356295 10.1093/nar/gkh824 PMC519115

[11] Matranga C, Tomari Y, Shin C, Bartel DP, Zamore PD. Passenger-strand cleavage facilitates assembly of siRNA into Ago2-containing RNAi enzyme complexes. Cell. 2005;123(4):607–20. doi: 10.1016/j.cell.2005.08.044 16271386 10.1016/j.cell.2005.08.044

[12] Tian B, Bevilacqua PC, Diegelman-Parente A, Mathews MB. The double-stranded-RNA-binding motif: Interference and much more. Nat Rev Mol Cell Biol. 2004;5(12):1013–23. doi: 10.1038/nrm1528 15573138 10.1038/nrm1528

[13] Ryter JM, Schultz SC. Molecular basis of double-stranded RNA-protein interactions: structure of a dsRNA-binding domain complexed with dsRNA. EMBO J. 1998;17(24):7505–13. doi: 10.1093/emboj/17.24.7505 9857205 10.1093/emboj/17.24.7505 PMC1171094

[14] Haase AD, Jaskiewicz L, Zhang H, Laine S, Sack R, Gatignol A, et al TRBP, a regulator of cellular PKR and HIV-1 virus expression, interacts with Dicer and functions in RNA silencing. EMBO Rep. 2005;6(10):961–7. doi: 10.1038/sj.embor.7400509 16142218 10.1038/sj.embor.7400509 PMC1369185

[15] Lee Y, Hur I, Park SY, Kim YK, Suh MR, Kim VN. The role of PACT in the RNA silencing pathway. EMBO J. 2006;25(3):522–32. doi: 10.1038/sj.emboj.7600942 16424907 10.1038/sj.emboj.7600942 PMC1383527

[16] Laraki G, Clerzius G, Daher A, Melendez-Pena C, Daniels S, Gatignol A. Interactions between the double-stranded RNA-binding proteins TRBP and PACT define the Medipal domain that mediates protein-protein interactions. RNA Biol. 2008;5(2):92–103. 18421256 10.4161/rna.5.2.6069

[17] Wilson RC, Tambe A, Kidwell MA, Noland CL, Schneider CP, Doudna JA. Dicer-TRBP complex formation ensures accurate mammalian microRNA biogenesis. Mol Cell. 2015;57(3):397–407. doi: 10.1016/j.molcel.2014.11.030 25557550 10.1016/j.molcel.2014.11.030 PMC4320653

[18] Wostenberg C, Quarles KA, Showalter SA. Dynamic Origins of Differential RNA Binding Function in Two dsRBDs from the miRNA "Microprocessor" Complex. Biochemistry. 2010;49(50):10728–36. doi: 10.1021/bi1015716 21073201 10.1021/bi1015716 PMC3565223

[19] Mueller GA, Miller MT, Derose EF, Ghosh M, London RE, Hall TM. Solution structure of the Drosha double-stranded RNA-binding domain. Silence. 2010;1(1):2 doi: 10.1186/1758-907X-1-2 20226070 10.1186/1758-907X-1-2 PMC2836000

[20] Du Z, Lee JK, Tjhen R, Stroud RM, James TL. Structural and biochemical insights into the dicing mechanism of mouse Dicer: a conserved lysine is critical for dsRNA cleavage. Proc Natl Acad Sci USA. 2008;105(7):2391–6. doi: 10.1073/pnas.0711506105 18268334 10.1073/pnas.0711506105 PMC2268147

[21] Sohn SY, Bae WJ, Kim JJ, Yeom KH, Kim VN, Cho Y. Crystal structure of human DGCR8 core. Nat Struct Mol Biol. 2007;14(9):847–53. doi: 10.1038/nsmb1294 17704815 10.1038/nsmb1294

[22] Yang SW, Chen HY, Yang J, Machida S, Chua NH, Yuan YA. Structure of arabidopsis HYPONASTIC LEAVES1 and its molecular implications for miRNA processing. Structure. 2010;18(5):594–605. doi: 10.1016/j.str.2010.02.006 20462493 10.1016/j.str.2010.02.006 PMC3119452

[23] Wostenberg C, Lary JW, Sahu D, Acevedo R, Quarles KA, Cole JL, et al The role of human Dicer-dsRBD in processing small regulatory RNAs. PLoS One. 2012;7(12):e51829 doi: 10.1371/journal.pone.0051829 23272173 10.1371/journal.pone.0051829 PMC3521659

[24] Lipari G, Szabo A. Model-Free Approach to the Interpretation of Nuclear Magnetic Resonance Relaxation in Macromolecules. 1. Theory and Range of Validity. J Am Chem Soc. 1982;104:4546–59.

[25] Mandel AM, Akke M, Palmer AG 3rd. Backbone dynamics of Escherichia coli ribonuclease HI: correlations with structure and function in an active enzyme. J Mol Biol. 1995;246(1):144–63. 7531772 10.1006/jmbi.1994.0073

[26] Brüschweiler R, Liao XB, Wright PE. Long-Range Motional Restrictions in a Multidomain Zinc-Finger Protein from Anisotropic Tumbling. Science. 1995;268(5212):886–9. 7754375 10.1126/science.7754375

[27] Lee LK, Rance M, Chazin WJ, Palmer AG 3rd. Rotational diffusion anisotropy of proteins from simultaneous analysis of 15N and 13C alpha nuclear spin relaxation. J Biomol NMR. 1997;9(3):287–98. 9204557 10.1023/a:1018631009583

[28] Quarles KA, Chadalavada D, Showalter SA. Deformability in the cleavage site of primary microRNA is not sensed by the double-stranded RNA binding domains in the microprocessor component DGCR8. Proteins: Struct Funct Bioinf. 2015;83(6):1165–79. doi: 10.1002/prot.24810 25851436 10.1002/prot.24810 PMC4446130

[29] Bevilacqua PC, Cech TR. Minor-groove recognition of double-stranded RNA by the double-stranded RNA-binding domain from the RNA-activated protein kinase PKR. Biochemistry. 1996;35(31):9983–94. doi: 10.1021/bi9607259 8756460 10.1021/bi9607259

[30] Ucci JW, Kobayashi Y, Choi G, Alexandrescu AT, Cole JL. Mechanism of interaction of the double-stranded RNA (dsRNA) binding domain of protein kinase R with short dsRNA sequences. Biochemistry. 2007;46(1):55–65. doi: 10.1021/bi061531o 17198375 10.1021/bi061531o

[31] Stephens OM, Haudenschild BL, Beal PA. The binding selectivity of ADAR2's dsRBMs contributes to RNA-editing selectivity. Chem Biol. 2004;11(9):1239–50. doi: 10.1016/j.chembiol.2004.06.009 15380184 10.1016/j.chembiol.2004.06.009

[32] Stefl R, Oberstrass FC, Hood JL, Jourdan M, Zimmermann M, Skrisovska L, et al The solution structure of the ADAR2 dsRBM-RNA complex reveals a sequence-specific readout of the minor groove. Cell. 2010;143(2):225–37. doi: 10.1016/j.cell.2010.09.026 20946981 10.1016/j.cell.2010.09.026 PMC2956598

[33] Quarles KA, Sahu D, Havens MA, Forsyth ER, Wostenberg C, Hastings ML, et al Ensemble analysis of primary microRNA structure reveals an extensive capacity to deform near the Drosha cleavage site. Biochemistry. 2013;52(5):795–807. doi: 10.1021/bi301452a 23305493 10.1021/bi301452a PMC3565094

[34] Acevedo R, Orench-Rivera N, Quarles KA, Showalter SA. Helical Defects in MicroRNA Influence Protein Binding by TAR RNA Binding Protein. PLoS One. 2015;10(1):e0116749 doi: 10.1371/journal.pone.0116749 25608000 10.1371/journal.pone.0116749 PMC4301919

[35] Parker GS, Maity TS, Bass BL. dsRNA binding properties of RDE-4 and TRBP reflect their distinct roles in RNAi. J Mol Biol. 2008;384(4):967–79. doi: 10.1016/j.jmb.2008.10.002 18948111 10.1016/j.jmb.2008.10.002 PMC2605707

[36] Roth BM, Ishimaru D, Hennig M. The core microprocessor component DiGeorge syndrome critical region 8 (DGCR8) is a nonspecific RNA-binding protein. J Biol Chem. 2013;288(37):26785–99. doi: 10.1074/jbc.M112.446880 23893406 10.1074/jbc.M112.446880 PMC3772224

[37] Huang Y, Ji L, Huang Q, Vassylyev DG, Chen X, Ma JB. Structural insights into mechanisms of the small RNA methyltransferase HEN1. Nature. 2009;461(7265):823–7. doi: 10.1038/nature08433 19812675 10.1038/nature08433 PMC5125239

[38] Masliah G, Barraud P, Allain FH. RNA recognition by double-stranded RNA binding domains: a matter of shape and sequence. Cell Mol Life Sci. 2013;70(11):1875–95. doi: 10.1007/s00018-012-1119-x 22918483 10.1007/s00018-012-1119-x PMC3724394

[39] Nguyen TA, Jo MH, Choi YG, Park J, Kwon SC, Hohng S, et al Functional Anatomy of the Human Microprocessor. Cell. 2015;161(6):1374–87. doi: 10.1016/j.cell.2015.05.010 26027739 10.1016/j.cell.2015.05.010

[40] Gleghorn ML, Maquat LE. 'Black sheep' that don't leave the double-stranded RNA-binding domain fold. Trends Biochem Sci. 2014;39(7):328–40. doi: 10.1016/j.tibs.2014.05.003 24954387 10.1016/j.tibs.2014.05.003 PMC4077987

